# Predictors of high school dropout, anxiety, and depression in genetic generalized epilepsy

**DOI:** 10.1002/epi4.12434

**Published:** 2020-09-24

**Authors:** Marte Syvertsen, Samitha Vasantharajan, Thea Moth, Ulla Enger, Jeanette Koht

**Affiliations:** ^1^ Department of Neurology Drammen Hospital Vestre Viken Hospital Trust Drammen Norway; ^2^ Institute of Clinical Medicine University of Oslo Oslo Norway

**Keywords:** education, epilepsy care, HADS, stigma

## Abstract

Affective disorders are overrepresented in epilepsy, and people with epilepsy may be at risk of dropping out from school. The aim of the present study was to assess factors influencing high school dropout, anxiety, and depression in genetic generalized epilepsy (GGE). One hundred and ten people with GGE aged 19‐40 years underwent a clinical interview, including the Hospital Anxiety and Depression Scale (HADS) questionnaire. Potential predictors of high school dropout were analyzed with logistic regression, and factors influencing total HADS score were analyzed with linear regression. Having felt excluded because of epilepsy was significantly associated with high school dropout (odds ratio 7.80, *P* = .009), as was total HADS score (odds ratio 1.22, *P* = .005). If a participant was currently employed or undergoing education, previous high school dropout was less likely (odds ratio 0.07, *P* = .005). High school dropout was associated with increased current anxiety and depression (*β* = 0.32, *P* = .005). Epilepsy severity (current drug resistance, current polytherapy, and active generalized tonic‐clonic seizures) was not associated with high school dropout, nor with total HADS score. The issue of stigma in epilepsy must be thoroughly addressed in comprehensive care and may be as important as seizure control when it comes to education and quality of life.

## 1 INTRODUCTION

Epilepsy is one of the most common neurological disorders, affecting approximately 0.7% of the population in the Nordic countries.^1^, ^2^ People with epilepsy suffer from sudden and unpredictable seizures, and affective disorders are overrepresented, with a strong association to reduced quality of life.^3^

Epileptic seizures may commence at any age, and nearly half of those affected are younger than 40 years.^4^ Adolescence is a vulnerable phase, when identity, acceptance, and belonging are major concerns. It is also a phase involving important choices, like education and future work. In addition to medical concerns, people with epilepsy are often faced with other people's anxiety and concerns regarding seizures, which may give rise to stigma and exclusion.^5^, ^6^, ^7^ Being diagnosed with epilepsy in adolescence represents a negative influence and may increase the risk of dropout. The exact reasons for dropout are unclear, however. They are nevertheless important to identify, in order to develop targeted preventive strategies.

The aim of the present study was to investigate predictors of anxiety, depression, and high school dropout in people affected by genetic generalized epilepsy (GGE) in adolescence.

## 2 METHODS

The study was cross‐sectional and hospital‐based. It was conducted at Drammen Hospital, which serves a population of 477 000 people in the southeast Norway. The hospital has no tertiary or otherwise specialized function in epilepsy care. Participants were identified by means of a systematic search of the hospital's medical records containing a diagnostic code of epilepsy (G40, International Classification of Diseases, 10th revision) in the period 1999‐2013.^4^ Additionally, participants were recruited consecutively from the electroencephalogram (EEG) laboratory at Drammen Hospital in the period 2014‐2018. Patients with GGE aged 19‐40 years who agreed to participate in a clinical interview were included in the study.

Childhood absence epilepsy (CAE), juvenile absence epilepsy (JAE), juvenile myoclonic epilepsy (JME), and epilepsy with generalized tonic‐clonic seizures only (EGTCS) were included in the definition of genetic generalized epilepsy (GGE), as recommended by the International League Against Epilepsy (ILAE).^8^ CAE, JAE, and EGTCS were defined according to the classification of the ILAE.^9^ JME was defined according to the class II diagnostic criteria of the consensus on diagnosis and management of JME.^10^ Patients with CAE who were seizure‐free for more than one year and not using antiepileptic drugs were excluded, as we wished to include only patients affected by epilepsy in adolescence.

The interviews took place from November 2016 to February 2018 and were conducted in a one‐to‐one setting, independent of regular clinical follow‐up. A semistructured questionnaire was used, including questions regarding background, medical history, and psychosocial issues. Stigma was addressed by asking participants if they ever felt excluded because of epilepsy (Appendix S1).

Additionally, the Hospital Anxiety and Depression Scale (HADS) questionnaire was administered to all participants. HADS consists of 14 questions, regarding present (within the last week) anxiety and depression. Minimum total score is 0, and maximum total score is 42. Higher scores indicate more anxiety and depression.^11^

The Norwegian educational system consists of ten years of compulsory school (age 6‐16 years), after which proceeding to three years of high school is optional (age 16‐19 years). Following graduation from high school, you can apply for admission to higher education (university or college). Dropout from high school was defined as having started the three‐year course of high school, but not having graduated.

Statistical analyses were performed using the Statistical Package for the Social Sciences (SPSS) software, version 25. Potential predictors of high school dropout were analyzed in a binary logistic regression model, and the different predictors’ influence on total HADS score was analyzed in a standard linear regression model. Preliminary analyses were conducted to ensure no violation of the assumptions of normality, linearity, multicollinearity, and homoscedasticity. *P*‐values < .05 were considered statistically significant.

The study was approved by the Regional Committee of Medical Research Ethics (reference 2013/1027) and by the data protection officer at Drammen Hospital. Written informed consent was obtained from all study participants.

## 3 RESULTS

One hundred and ten people with GGE aged 19‐40 years were included in the study. Demographic, clinical, and psychosocial variables are reported in Table 1. Three patients (3%) did not proceed to high school after finishing ten years of compulsory schooling. Of the 107 patients who started high school, 19 (18%) did not graduate.

**TABLE 1:** Demographic, clinical, and psychosocial variables of the 110 study participants

Female	62 (56%)
Mean age at inclusion (y)	27 ± 6
Mean epilepsy duration (y)	13 ± 7
Childhood absence epilepsy (CAE)	7 (6%)
Juvenile absence epilepsy (JAE)	15 (14%)
Juvenile myoclonic epilepsy (JME)	77 (70%)
Epilepsy with generalized tonic‐clonic seizures only (EGTCS)	11 (10%)
Current polytherapy	20 (18%)
GTCS within the last year	21 (19%)
Current drug‐resistant epilepsy	22 (20%)
Currently working or studying	86 (78%)
Felt excluded because of epilepsy	22 (20%)
Wished to keep diagnosis of epilepsy secret	45 (41%)
Troublesome conditions at home	35 (32%)
Use of mental health services	57 (20%)
Epilepsy influenced the choice of education and/or work	47 (43%)
Diagnosed with ADHD	8 (3%)
High school dropout	19 (17%)
Highest educational level	
Secondary school (10 years)	22 (8%)
High school (13 years)	58 (20%)
University/college education of <4‐year duration	21 (7%)
University/college education of >4‐year duration	9 (3%)

Abbreviation: ADHD, attention‐deficit/hyperactivity disorder.

A binary logistic regression model was used to assess the impact of different predictors on the likelihood of high school dropout. Independent variables included were as follows: age, gender, current drug resistance (as defined by the ILAE),^12^ current polytherapy (use of ≥two antiepileptic drugs), GTCS within the last year, having felt excluded because of epilepsy, currently working or studying, wishing to keep the diagnosis secret, troublesome conditions at home (parent(s) with psychosocial problems like addiction or violent behavior), being diagnosed with attention‐deficit/hyperactivity disorder (ADHD), use of mental health services, and total HADS score. The model was statistically significant (*P* < .001), explaining between 34.5% (Cox & Snell R Square) and 57.2% (Nagelkerke R Square) of the variance of the dependent variable (high school dropout). The variables significantly associated with high school dropout were current work or education (odds ratio 0.07), having felt excluded because of epilepsy (odds ratio 7.80), wishing to keep the diagnosis secret (odds ratio 0.17), and total HADS score (odds ratio 1.22), Table 2. A standard linear regression model was used to assess moderators of total HADS score. Independent variables included were as follows: age, gender, current drug resistance, current polytherapy, GTCS within the last year, having felt excluded because of epilepsy, currently working or studying, wishing to keep the diagnosis secret, troublesome conditions at home, high school dropout, being diagnosed with ADHD, and use of mental health services. The model was statistically significant (*P* = .013), explaining 22.3% of the variance in total HADS score. Dropout from high school was the only significant moderator of total HADS score (*β* = 0.32, *P* = .005).

**TABLE 2:** Predictors of high school dropout in genetic generalized epilepsy

	*P*‐value	OR	95% confidence interval for OR
Gender	0.201	3.04	0.55‐16.71
Age	0.28	0.93	0.82‐1.06
Current drug‐resistant epilepsy	0.05	5.94	0.97‐36.5
Current polytherapy	0.63	0.63	0.09‐4.25
GTCS within the last year	0.18	3.26	0.58‐18.16
Currently working or studying	0.005*	0.07	0.01‐0.45
Felt excluded because of epilepsy	0.009*	7.80	1.67‐36.47
Wished to keep diagnosis of epilepsy secret	0.046*	0.170	0.03‐0.97
Total HADS score	0.005*	1.23	1.06‐1.42
Troublesome conditions at home	0.193	3.26	0.55‐19.21
Use of mental health services	0.77	0.76	0.12‐4.99
Diagnosed with ADHD	0.153	6.97	0.47‐99.90

Abbreviation: OR, odds ratio.

* Statistically significant.

## 4 DISCUSSION

In the present study, having felt excluded because of epilepsy was significantly associated with dropout from high school, whereas epilepsy severity was not. High school dropout was also associated with current anxiety and depression, whereas epilepsy severity was not. People who were employed or studying at the time of the interview were less likely to have dropped out from high school. We were not able to identify previous studies analyzing predictors of school dropout in epilepsy. Still, this is a highly important issue, as we have demonstrated by the expected association between dropout and current employment.

A recent Norwegian study interviewed 96 young people (mean age 24 years) at risk of early work disability.^13^ Sixty‐six percent confirmed that they had experienced bullying. The authors conclude that psychological and relational factors are important when it comes to vocational rehabilitation, in line with our finding of perceived exclusion as a predictor of high school dropout.

Although reasons for school dropout in epilepsy have not been much discussed, it is well known that stigma and social challenges are associated with this diagnosis.^5^ Twenty percent of the participants in the present study reported that they had felt excluded because of epilepsy. In a recent study by Gabriel et al, 28% of 226 people with GGE felt stigmatized. Fifty‐one (22%) were unemployed, which was more than the number of people with refractory epilepsy (n = 39, 17%).^7^

In the present study, severity of the epilepsy did not significantly influence the presence of affective symptoms, nor high school dropout, although current drug resistance came very close, at a *P*‐value of .05. Similarly, an older study from Finland found increased risk of social and educational problems in a long‐term follow‐up study of 245 children with epilepsy, even though the majority were seizure‐free.^14^ Hence, educational and occupational problems in epilepsy do not seem to be strictly related to seizure control, and stigma may be a significant contributing factor.

Information and dialogue are efficient measures in the prevention of stigma.^15^ Yet, several authors state that even people with epilepsy lack substantial knowledge about their own diagnosis.^16^, ^17^, ^18^ In a survey of 1182 visitors at the web page of the Norwegian Epilepsy Association in 2017, the majority of people interested in psychosocial issues reported that they did not get the information they wanted, and more than 90% wished for more general information concerning epilepsy.^19^ This demonstrates that we still have a long way to go when it comes to epilepsy awareness and knowledge, even in a country with relatively high educational level and standard of living, in a time when any kind of information is accessible online. Our findings call for targeted and accessible information, in addition to improved dialogue between epilepsy expertise and local caregivers.

The present study was based on retrospective self‐report from a limited dataset not verified by public records, which is a considerable limitation. Still, as data regarding reasons for school dropout in epilepsy are lacking, we provide valuable clues for the focus of future investigations and preventive work. Seizure freedom cannot be obtained for all, but psychosocial issues and stigma/exclusion may be just as important when it comes to dropout from school.

Participants were asked whether they ever felt excluded because of epilepsy, but a validated questionnaire regarding stigma was not used. Such a questionnaire would have been a more reliable tool. Retrospectively asking about perceived exclusion because of epilepsy may be influenced by recall bias and by current mood. Hence, HADS score was useful in adjustment for current mood in the regression analysis of factors influencing high school dropout.

Even though HADS investigates symptoms during the last week, it is considered a reliable tool when it comes to identifying cases of anxiety and depression (Bjelland 2002).^20^ Current HADS score was significantly associated with previous dropout from high school. Stimulating and meaningful work environments represent protective factors when it comes to mood disorders. Less stimulating work environments do not necessarily have the same protective effect.^21^, ^22^ We found no association between HADS score and whether the patient was currently working/studying or not. We did find an association between HADS score and high school dropout, however. This could reflect that patients who did not graduate from high school were unable to benefit from the stimulating and protective work environments higher education gives access to.

We find it interesting that there was no relation between current anxiety and depression and the presence of GTCS, nor with current polytherapy or drug resistance. However, the regression model analyzing HADS moderators explained only 22% of the total variance in HADS score, meaning that other, significantly influential factors remain to be identified. The regression model analyzing high school dropout was a good fit, on the other hand, explaining up to 57% of the variance in educational status (high school dropout or not). Still, other epilepsy‐related factors potentially contributing to dropout from school were not addressed in the present study. Epilepsy is associated with cognitive challenges, including difficulties regarding attention, processing speed, and memory, and academic underachievement is common in children with active epilepsy.^23^, ^24^, ^25^, ^26^ All in all, school may be perceived as more effortful to children and adolescents with epilepsy, increasing the risk of dropout.

Another limitation to consider is that only people with GGE were included. This represents a bias, since a specific behavioral profile has been suggested in this group of patients, more precisely in JME.^27^ The majority of our participants were diagnosed with JME. Disinhibited behavior possibly caused by subtle frontal lobe dysfunction could contribute to stigma and exclusion and possibly account for some of the variance related to both high school dropout and HADS score. Nevertheless, GGE is a rather homogenous group, without the intellectual and/or physical disability that may be related to other types of epilepsy, and which could be more evident triggers of stigma and exclusion.

We did not have information about treatment, drug resistance, or seizure activity during school years, which is a limitation. However, the majority of study participants had JME. The long‐term clinical course of JME seems to be stable and may even improve at older age.^28^, ^29^

When it comes to evaluation of current seizure freedom, only GTCS within the last year was considered, as myoclonic jerks and absences may be subtle and sometimes go by unnoticed. Moreover, GTCS is a more dramatic seizure type, with more extensive social (and physical) consequences than myoclonic jerks and absences.

We have demonstrated an association between high school dropout and current affective symptoms, and we have identified perceived exclusion because of epilepsy as an important predictor of high school dropout. School dropout influences ability to work and study later in life, and affective disorders are strongly linked to quality of life in epilepsy.^3^ If stigma causes school dropout, and school dropout is linked to unemployment, affective symptoms, and reduced quality of life, addressing the issue of stigma at an early stage in epilepsy seems extremely important. Due to the retrospective nature of our study and limitations like the lack of a validated questionnaire to assess stigma, the mentioned associations must be interpreted with great care, however. Nevertheless, we hope to have raised important questions regarding the influence of stigma on future education and work. These important issues should be addressed in larger and preferably prospective studies.

## CONFLICTS OF INTEREST

Dr Syvertsen has received speaker honoraria from Eisai. The remaining authors have no conflicts of interest. We confirm that we have read the Journal's position on issues involved in ethical publication and affirm that this report is consistent with those guidelines.

## Supporting information

Supplementary Material
