# Supplementary material for: Predictors of high school dropout, anxiety, and depression in genetic generalized epilepsy
Source: Epilepsia Open. 2020 Sep 24;5(4):611–5. doi: 10.1002/epi4.12434 (PMC7733661; doi:10.1002/epi4.12434)
Supplement: Supplementary file 1 — Supplementary Material [file EPI4-5-611-s001.doc]

**SEMI-STRUCTURED INTERVIEW**

**GENETIC GENERALIZED EPILEPSY (GGE)**

**Background**

**1. Gender** ❑ Female ❑ Male

**2. Age: __________ years**

**3. Do any other members of the family have epilepsy?**

❑Mother/father

❑Sibling

❑Children

❑Grandparent

**4. Does the patient have children of his/her own?**

❑Yes ❑No

**5. Did the patient receive child welfare services?**

❑Foster care ❑Other ❑Unknown

**6. Did the patient’s child(ren) receive child welfare services?**

❑Foster care ❑Other ❑Unknown

**7. Was the patient offered special education at school?**

❑Yes ❑No ❑Unknown

**8. Does the patient think that he/she has been much away from school?**

❑Yes ❑No ❑Unknown

**9. Did the patient ever start high school?**

❑Yes ❑No ❑Unknown

**10. Highest level of completed education**

❑Elementary school (7 years)

❑Secondary school (10 years)

❑High school (13 years)

❑College/university < 4 years

❑College/university > 4 yeras

**11. Current work/education**

❑Employed

❑Self-employed

❑Under education

❑Unemployed and not under education, receiving social services

❑Unemployed and not under education, not receiving social services

**12. Did the epilepsy influence choice of education and/or work?**

❑Yes ❑No ❑Unknown

**Epilepsy**

**13. Seizure types**

❑Myoclonic jerks

❑Absences

❑GTCS

**14. Age at seizure onset**

Myoclonic jerks:______________

Absences:________________

GTCS:___________

**15. Age at initiation of treatment with antiepileptic drugs**

________________________

**16. Seizure frequency myoclonic jerks**

❑Daily

❑Weekly

❑Monthly

❑Less than monthly

❑Seizure free for more than one year

❑Never had myoclonic jerks

**17. Seizure frequency absences**

❑Daily

❑Weekly

❑Monthly

❑Less than monthly

❑Seizure free for more than one year

❑Never had absences

**18. Seizure frequency GTCS**

❑Daily

❑Weekly

❑Monthly

❑Less than monthly

❑Seizure free for more than one year

❑Never had GTCS

**19. What was the year of the last seizure?**

Myoclonic jerks:___________________

Absences:____________________

GTCS:________________________

**20. At what time of the day do myoclonic jerks occur?**

❑Mornings ❑After sleep in general (including naps)

❑Daytime ❑Afternoon/evening

❑At night ❑The patient does not have myoclonic jerks

**21. At what time of the day do absences occur?**

❑Mornings ❑After sleep in general (including naps)

❑Daytime ❑Afternoon/evening

❑At night ❑The patient does not have absences

**22. At what time of the day do GTCS occur?**

❑Mornings ❑After sleep in general (including naps)

❑Daytime ❑Afternoon/evening

❑At night ❑The patient does not have GTCS

**23. Does the patient feel particularly tired or unwell in the morning?**

❑Yes ❑No ❑Unknown

**Antiepileptic drugs**

**24. Number of antiepileptic drugs in current use**

❑None

❑One

❑Two

❑More than two

**25. Response to treatment**

❑Seizure free due to lifestyle-changes, without antiepileptic drugs

❑Seizure free using one antiepileptic drug

❑Seizure free using two or more antiepileptic drugs

❑Drug resistant

❑Pseudorefractory

**26. Which antiepileptic drugs are currently in use?** *(Name + dose)*

**27. Which antiepileptic drugs have been tried previously?** *(Name + dose)*

**28. Other drugs currently in use** *(Name + dose)*

**29. Did the patient ever try to stop antiepileptic medication?**

❑Yes ❑No

**If yes, what was the result?**

❑Stayed seizure free without antiepileptic drugs

❑Recurrence of seizures, but did not restart antiepileptic drug treatment

❑Recurrence of seizures, restarted antiepileptic drug treatment and became seizure free

❑Recurrence of seizures, restarted antiepileptic drug treatment but was refractory

**Who decided to stop antiepileptic drug treatment?**

❑The patient, without consulting a doctor

❑The patient asked a doctor

❑The doctor suggested it to the patient

**For how long was the patient seizure free prior to stopping antiepileptic drug treatment?**

❑0-2 years

❑2-5 years

❑5-10 years

❑More than 10 years

**Psychosocial aspects**

**30. Has there been need of psychiatric health care?**

❑Yes ❑No ❑Unknown

**31. Was ADHD suspected?**

❑Examined for ADHD

❑Diagnosed with ADHD

❑No

❑Unknown

**32. Did the patient ever experience a psychosis?**

❑Yes ❑No ❑Unknown

**33. Did the patient ever feel excluded because of epilepsy?**

❑Yes ❑No ❑Unknown

**34. Has the patient avoided any social activities because of epilepsy?**

❑Yes ❑No ❑Unknown

**35. Has the patient experience bullying?**

❑Yes ❑No ❑Unknown

**36. Did the patient ever wish to keep the diagnosis of epilepsy a secret?**

❑Yes ❑No ❑Unknown

**37. Current or previous suicidal thoughts**

❑Yes ❑No ❑Unknown

**38. Current or previous self-mutilation**

❑Yes ❑No ❑Unknown

**39. Parents with psychosocial challenges like addiction or violent behavior**

❑Yes ❑No ❑Unknown

**40. Victim of violence or abuse**

❑Yes ❑No ❑Unknown

**41. Unplanned pregnancy**

❑Yes ❑No ❑Unknown

**42. Use of illicit recreational drugs on more than two occasions**

❑Yes ❑No ❑Unknown

**43. Smoking cigarettes prior to the age of 18 years**

❑Yes ❑No ❑Unknown

**44. Police -charges**

❑Yes ❑No ❑Unknown
